# Therapeutic Prospects of Mesenchymal Stem Cell and Their Derived Exosomes in the Regulation of the Gut Microbiota in Inflammatory Bowel Disease

**DOI:** 10.3390/ph17050607

**Published:** 2024-05-09

**Authors:** Yaru Qiao, Xiaohua Tang, Ziyue Liu, Dickson Kofi Wiredu Ocansey, Mengjiao Zhou, Anquan Shang, Fei Mao

**Affiliations:** 1 Key Laboratory of Medical Science and Laboratory Medicine of Jiangsu Province, School of Medicine, Jiangsu University, Zhenjiang 212013, China; 3200902006@stmail.ujs.edu.cn (Y.Q.); 15952860512@163.com (Z.L.); dickson.ocansey@ucc.edu.gh (D.K.W.O.); zmjssll@163.com (M.Z.); 2 Department of Laboratory Medicine, Lianyungang Clinical College, Jiangsu University, Lianyungang 222006, China; shanganquan@tongji.edu.cn; 3 The People’s Hospital of Danyang, Affiliated Danyang Hospital of Nantong University, Zhenjiang 212300, China; gzjj0808@163.com; 4 Department of Medical Laboratory Science, School of Allied Health Sciences, College of Health and Allied Sciences, University of Cape Coast, Cape Coast CC0959347, Ghana

**Keywords:** mesenchymal stem cells, exosomes, inflammatory bowel disease, gut microbiota, therapy

## Abstract

Mesenchymal stem cells (MSCs) have shown great potential in the treatment of several inflammatory diseases due to their immunomodulatory ability, which is mediated by exosomes secreted by MSCs (MSC-Exs). The incidence of inflammatory bowel disease (IBD) is increasing globally, but there is currently no long-term effective treatment. As an emerging therapy, MSC-Exs have proven to be effective in alleviating IBD experimentally, and the specific mechanism continues to be explored. The gut microbiota plays an important role in the occurrence and development of IBD, and MSCs and MSC-Exs can effectively regulate gut microbiota in animal models of IBD, but the mechanism involved and whether the outcome can relieve the characteristic dysbiosis necessary to alleviate IBD still needs to be studied. This review provides current evidence on the effective modulation of the gut microbiota by MSC-Exs, offering a basis for further research on the pathogenic mechanism of IBD and MSC-Ex treatments through the improvement of gut microbiota.

## 1. Introduction

Mesenchymal stem cells (MSCs) are a type of adult stem cell that can differentiate into various cell types, such as bone, cartilage, fat, and muscle cells. MSCs have been widely studied for their therapeutic potential in regenerative medicine due to their ability to promote tissue repair and modulate the immune system [1,2]. Extracellular vesicles, including exosomes released by MSCs (MSC-Exs), carry various biological molecules such as proteins, lipids, and nucleic acids like microRNAs. Exosomes can act as messengers between cells, delivering their cargo to target cells and influencing their behavior. MSC-Exs have shown potential in promoting tissue repair, modulating the immune response, and inhibiting inflammation, making them a promising therapeutic tool in regenerative medicine and immune-related disorders, including inflammatory bowel disease (IBD) [3,4].

The gut microbiota refers to the community of microorganisms that live in the gastrointestinal tract, including bacteria, viruses, fungi, and other microbes. The gut microbiota plays a vital role in maintaining the health of the host by aiding digestion, producing vitamins, and protecting against pathogenic bacteria [5]. IBD, a chronic inflammatory disorder of the gastrointestinal tract, includes Crohn’s disease (CD) and ulcerative colitis (UC). The exact cause of IBD is not fully understood, but it is believed to be due to a complex interplay between genetic, environmental, and microbial factors [6]. Emerging evidence suggests that dysbiosis, an imbalance in the gut microbiota, may play a critical role in the development and progression of IBD. Changes in the composition of the gut microbiota, such as a decrease in beneficial bacteria or an increase in harmful bacteria, have been observed in patients with IBD [7,8]. In addition, the gut microbiota can lead to an inappropriate immune response, resulting in chronic inflammation in the gut, and can produce metabolites that affect the gut environment and immune system [9].

Therefore, the gut microbiota is considered a potential therapeutic target for the treatment of IBD. Strategies such as probiotics, prebiotics, and fecal microbiota transplantation (FMT) are being investigated for their ability to restore gut microbial balance and alleviate inflammation in IBD. Other therapeutic agents such as MSCs and MSC-Exs have attracted research attention in this field since studies have reported their role in regulating the gut microbiota in IBD by altering the abundance of specific bacterial species, leading to a reduction in proinflammatory bacteria and an increase in anti-inflammatory bacteria [10]. This study examines current evidence on the regulatory role of MSCs and MSC-Exs on the gut microbiota in IBD and provides a basis for further research.

## 2. Gut Microbiota and IBD

### 2.1. Composition of Gut Microbiota and Its Influencing Factors

**Figure 1:**
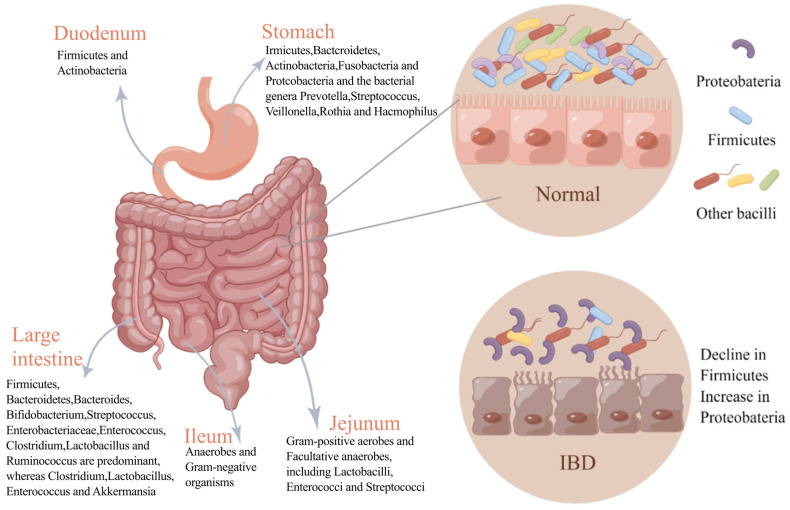
Changes in gut microbiota in normal and IBD states. The figure depicts the bacterial composition of different parts of the normal gastrointestinal tract and changes in the flora in states of intestinal inflammation. In intestinal inflammation, the diversity of the gut microbiota is reduced, and the dominant flora is altered compared with the healthy gut microbiota.

The gastrointestinal tract consists of three parts: the stomach, the small intestine, and the large intestine. Each of these parts has a different composition of microorganisms (Figure 1). For a long time, it was generally believed that the stomach has no bacterial growth due to its strong acidity, but after a lot of research, it was found that the stomach contains a large number of acid-resistant strains. Due to the development of culture-free techniques, five major phyla have been detected in the stomach: Firmicutes, Bacteroidetes, Actinobacteria, Fusobacteria, and Proteobacteria. Prevotella, Streptococcus, Veillonella, Rothia, and Haemophilus are the main flora in the healthy human stomach [11]. The small intestine (SI) can be divided into the duodenum, jejunum, and ileum, and different parts have different bacterial compositions and content due to their microenvironments. The duodenum predominantly contains Firmicutes and Actinomycetes; the jejunum supports the growth of Gram-positive aerobic and facultative anaerobes, including *Lactobacilli*, *Enterococci*, and *Streptococci*; and the ileum supports predominantly anaerobic and Gram-negative bacteria, similar to the colon [12]. Anaerobic bacteria mainly dominate the large intestine (LI) [13], and their ratio is closely related to the individual’s health status. At the same time, *Bacteroides*, *Bifidobacteria*, *Streptococcus*, *Enterobacteriaceae*, *Enterococcus*, *Clostridium*, *Lactobacillus*, and *Ruminococcus* are the major bacterial genera in the large intestine.

The gut microbiota is affected by many factors, which can be roughly divided into three categories: host endogenous factors, exogenous factors, and environmental factors [14]. Host endogenous factors include age. A study of genomic analyses (MetaOTUs) of the gut microbiota of infants one year after birth found that the diversity and number of gut microbiota increased as the infants aged; in contrast to the mother’s gut microbiota, the composition becomes more similar with age [15]. In a study on the fungal group in middle-aged and elderly people, the fungal composition significantly differed between the two groups. In the elderly population, *Germella* and *Ascystis* were absent, whereas *Malassezia* was absent in middle-aged individuals but abundant in the elderly [16]. Host exogenous factors include diet, drugs, and lifestyle. Diet changes the metabolites of gut microbiota through the influence of microorganisms, influencing health [17]. In addition, one study showed that vegetarian and omnivorous diets can increase fecal amino acid metabolites, where dietary fiber helps in the recovery of the human gut microbiome and its metabolome by promoting the growth of healthy bacteria populations [18]. Drugs can also affect the gut microbiota, and some of the microbiota may also reduce drug efficacy. Different geographical locations also affect the gut microbiota. In addition, temperature and some extreme conditions cause changes in the gut microbiota [12].

The adult gut microbiota is a large, diverse, and dynamic ecosystem. There are 100 trillion microorganisms in the human intestinal tract, which is ten times the total number of human cells, accounting for the vast majority of the total number of microorganisms in the human body, and they have an important impact on the physiological and pathological conditions of the human body [19]. A study on DNA sequencing of 124 stool samples in Europe aimed at adding to the gene catalog of human gut microbes found that almost all the genes came from bacteria; only 0.1% were of eukaryotic and viral origin, and the rest were of archaeal origin. In that study, there were a total of 1000 to 1150 bacterial species, with at least 160 bacterial species per individual, indicating that the gut microbiota has individual variability [20].

The gut microbiota protects the intestinal mucosa from pathogenic bacteria through its specific metabolites and immunomodulatory effects [21]. The normal microbiota is relatively stable and forms a “defense wall” to resist the colonization of abnormal microorganisms and the expansion of pathogenic bacteria. This phenomenon is referred to as the “Colonization Resistance” of microorganisms and is accomplished through direct or indirect mechanisms, including the production of inhibitory metabolites, the release of bactericidal substances, and competition for resources [22].

The immune function of the intestine is related to the gut microbiota. The composition of the neonatal gut microbiota is closely related to the composition of its immune cells. Children with higher *Bifidobacteria* content tend to have more anti-inflammatory T cells, and breastfeeding-obtained *Bifidobacteria* can inhibit the occurrence of intestinal inflammation [23]. The colonization of gut microbiota in infancy is closely related to the development and maturation of immune cells. Different microorganisms produce corresponding stem cell populations, and if antibiotics are used early in life, the number of stem cells that develop into immune cells (Paneth cells and macrophages) is reduced [24]. In addition to affecting immune cells, antibody production is also regulated by the gut microbiota. As the most abundant antibody in the intestinal mucosa, IgA plays an essential role in the immune defense of the intestinal tract. However, the secretion of IgA requires the induction of intestinal commensal bacteria. In the early stages of human life, before the colonization of the gut microbiota, IgA is lacking in the intestinal mucosa, confirming the crucial role of the gut microbiota in health and diseases [25].

### 2.2. IBD and Gut Microbiota Role

In recent years, IBD has become a global disease, and the incidence of IBD in newly industrialized countries such as those in Asia has been increasing [26]. In China, IBD has migrated from a rare disease to a common disease [27]. There is no doubt that IBD has become an important health problem globally. As a highly heterogeneous chronic inflammatory disease of the gastrointestinal tract, IBD exhibits varying severity and symptoms in different patients. IBD is often diagnosed in adolescence and early adulthood and usually presents with gastrointestinal symptoms such as abdominal pain, diarrhea, and bleeding, as well as systemic symptoms such as anemia and weight loss [28]. Extraintestinal manifestations can also occur, often involving the joints, skin, eyes, and other organs such as the liver, lungs, and pancreas [29]. In addition, IBD can also cause a series of complications, such as fistula, infection, and colorectal cancer [30]. IBD exhibits complex and refractory characteristics and is usually challenging to completely cure. IBD treatment aims to control the acute and progressive exacerbation of inflammation, maintain remission, and treat corresponding complications [31].

The interaction of multiple factors such as environment, genetics, immunity, and intestinal microorganisms causes IBD. The pathogenesis of IBD is very complicated and has not been fully understood. For a long time, the gut microbiota has been considered an important factor in the pathogenesis of IBD, and the diversity and quantity of gut microbiota in IBD patients are abnormal [32]. Most studies have demonstrated that a dysfunctional interaction between the gut microbiota and the immune response of the intestinal epithelium can cause IBD. However, the relationship between them is not clear, and a review showed that IBD is due to a genetic defect that makes the intestinal epithelial immune system abnormally responsive to gut microbes [33]. Human intestinal bacteria have been implicated in the pathogenesis of IBD [34]. For example, Actinobacillus *Eggerthella lenta* (*E. lenta*) can relieve the inhibition of Th cell transcription factors; activate proinflammatory cells and Th 17 cells; and trigger intestinal inflammation, and *E. lenta* strains are significantly enriched in IBD patients [35]. Moreover, transplanting *Klebsiella pneumonaea* strains from IBD patients into sterile colonized mice increases the probability of IBD in mice, and this effect can be effectively alleviated by *Klebsiella pneumonaea*-targeting phages [36]. In these studies, changing only gut microbes can enhance intestinal inflammation, indicating that microbes play an indispensable role in the development of IBD. In addition, the intestinal microbial composition of IBD patients is very different from that of healthy people. The diversity of gut microbiota in IBD patients is significantly decreased, with reduced Firmicutes and Bacteroidetes but increased Proteobacteria (Figure 1). The metabolism pathways and their associated functions are also correspondingly altered, and the bacterial network in IBD patients is also altered compared with normal people [37].

### 2.3. IBD Treatment: Microbiota Target

At present, IBD is commonly treated with drugs such as immunosuppressants, biological agents, and antibiotics, but these treatments have certain limitations. Recently, there has been increasing attention on the treatment of IBD by targeting the gut microbiota, such as fecal transplants and probiotic transplants. Fecal microbiota transplantation (FMT) introduces microbes from healthy donors’ feces into patients to improve intestinal microbial imbalance and achieve therapeutic effects [32]. An IBD study on combined *Clostridium difficile* infection (CDI) showed that FMT can significantly increase the diversity of intestinal microorganisms and significantly change the microbial composition, effectively treating CDI, but it easily recurs [38]. Harry Sokol et al., in the co-treatment of CD with cortisol and FMT, found that no patient reached the primary endpoint of the treatment, i.e., the colonization of the donor flora within the sixth week, and concluded that the colonization of the donor flora in CD may be associated with maintaining remission [39]. In a recent study [40], gut bacteria from UC patients and healthy donors (HD) were transplanted separately into a mouse model. The results showed that the two donor flora had a regulatory effect on the gut microbiota of the mice, and the HD bacteria group significantly reduced the expression of proinflammatory factors in the model mice, where the effect of improving IBD was better in HD than that of the UC bacteria group.

Intestinal probiotics mainly include Bifidobacteria and lactic acid bacteria. Probiotic transplantation can regulate gut microbiota disturbance, thereby reducing intestinal inflammation. A study showed that the fecal microbiota of IBD patients were rich in Enterobacteriaceae, and the transplantation of IBD patients’ fecal microbiota could cause colitis. The oral administration of *Lactobacillus plantarum NK151*, *Bifidobacterium longum NK173*, and *Bifidobacterium NK175* can alleviate colitis by inhibiting intestinal bacterial lipopolysaccharide (LPS) and regulating the expression of proinflammatory and anti-inflammatory cytokines [41]. Studies have shown that *Clostridium butyricum* (*C. butyricum*) protects the intestinal barrier and regulates the gut microbiota, in which EV produced by *Chlamydia butyricum* plays an important role [42]. Although fecal and probiotic transplants have been proven to be useful in the treatment of IBD, they have the disadvantages of easily recurring and uncertain efficacy. MSC is an emerging treatment method for IBD, and it may be an effective alternative therapy to alleviate IBD by regulating gut microbiota through MSC-Exs.

## 3. The Role of MSCs and MSC-Exs in the Regulation of IBD-Associated Gut Microbiota

**Figure 2:**
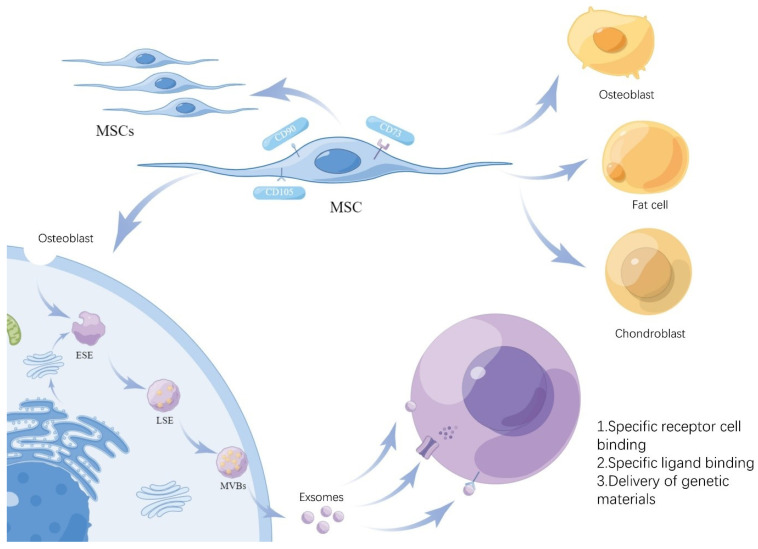
Formation of MSC-Exs. The diagram illustrates the ability of MSCs to self-replicate and differentiate and the key aspects of exosome formation. MSCs form ESEs through endocytosis or Golgi secretion and are secreted out of the cell in two stages, LSEs and MVBs, to form exosomes that act on target cells.

Mesenchymal stem cells (MSCs) are cells with multipotential differentiation potential that can be isolated from different tissues of humans and various mammals [43]. Human MSCs are mostly extracted from bone marrow, adipose tissue, and neonatal birth-associated tissues, including the placenta, amniotic fluid, and umbilical cord, and have the ability to self-replicate and differentiate into multiple lineages of mesenchyme [44,45] (Figure 2). In addition to the ability to differentiate multi-lineages and self-renew, MSCs also have the ability to regulate the immune system [46,47]. Therefore, MSCs are regarded as a possible treatment for many immune and inflammatory diseases, including IBD. MSCs have been proven to be effective in alleviating IBD, but the precise mechanisms by which they exert their effects within the intestinal microenvironment remain to be fully explored. It is hypothesized that their therapeutic impact may be attributable, at least in part, to the modulation of intestinal microbiota. Emerging evidence suggests that MSCs can significantly influence antimicrobial activities, directly and indirectly interacting with gut microorganisms [48].

Accumulating evidence indicates that MSC immunomodulation depends on the cells’ paracrine effects, including soluble factors and large numbers of extracellular vesicles (EVs). A variety of cell types secrete EVs and are also present in various body fluids [49]. The release of EVs contributes to intercellular communication. EVs are divided into three types: exosomes, microvesicles, and apoptotic bodies. Their sizes and origins are different. Among them, the most numerous EV subtypes are exosomes [50]. Exosomes originate from endosomes, where cell membranes form early endosomes (ESEs) through endocytosis and lipid depressions, which can also be promoted by the endoplasmic reticulum and Golgi apparatus, and ESEs can further mature into late endosomes (LSEs), and finally form multivesicular bodies (MVBs). MVBs fuse with cell membrane lipids and are released to form multiple exosomes with a diameter of 30–150 nm (Figure 2) [51,52].

Exosomes are composed of lipids, proteins, RNA, and DNA. Exosomes contain functionally rich proteins, among which, transmembrane proteins include CD9, CD63, CD81, and CD82, which are involved in cell penetration, invasion, and fusion events. The important RNA in exosomes is miRNA; it has less DNA content, which has a smaller effect. At the same time, exosomes contain different types of lipids [53]. Exosomes act on target cells by (1) specifically binding to target receptor cells to exchange proteins and lipids; (2) binding receptors to target ligands to trigger downstream signaling events; and (3) transferring genetic material through membrane fusion, receptor–ligand interactions, or endocytosis [54]. It is generally believed that the efficient delivery of genetic material is crucial for successfully applying exosomes. A number of studies have shown the superior therapeutic effect of MSC-Exs compared with MSCs, and they lack some side effects of MSC treatment, such as immune rejection. At the same time, MSC-Exs can also be used as carriers in nano-medicine [55]; therefore, the applications of MSC-Exs in disease treatment have received extensive attention.

### 3.1. MSCs and MSC-Exs in the Regulation of IBD-Associated Gut Flora

#### 3.1.1. Reduction in Harmful Flora

The gut microbiota of IBD patients show an abnormal increase in certain commensal flora, such as Proteobacteria, which are low in the intestines of healthy individuals and lead to the ecological dysregulation of the intestinal tract. According to research, D-amino acids can inhibit the growth of Proteobacteria. The administration of D-amino acids to experimental colitis mice can alleviate intestinal inflammation [56]. Another piece of evidence in a 16srRNA sequencing analysis of mouse feces showed that intestinal inflammation significantly increased the proportion of Proteobacteria, such as *Proteus* and Bacteroides, compared with a healthy group. However, the treatment of DSS-induced colitis mice with MSCs significantly restored microbiota alterations and inhibited the increase in Proteobacteria [57].

In addition, the normal intestinal tract flora, *E. coli*, which can be induced to overgrow by intestinal inflammation [58], and pathogenic *E. coli* also play an integral role in the progression of inflammation. Adherent invasive *E. coli* (AIEC) has been reported to be associated with intestinal epithelioid granulomas manifested by CD, whereas diffuse adherent *E. coli* (DAEC) is often isolated with the feces of patients with UC [59]. Recently, atypical enteropathogenic *E. coli* (a-EPEC) has also been found to be associated with laboratory and clinical UC [60]. The infusion of UC-MSCs reduces the Enterobacteriaceae family in the gut microbiota and protects against invasion by pathogenic *E. coli* [61]. MSCs normalize *E. coli* levels in the gut microbiota and help to alleviate intestinal inflammation.

*Fusobacterium varium* is normally found in the human oral cavity and can adhere to and invade intestinal epithelial cells. Fusobacteria has been isolated from the intestinal mucosa of patients with UC [62]. It has been found that enemas given to mice with supernatants obtained from culturing *Fusobacterium varium* resulted in the development of UC in these mice [63]. The secretome of dental pulp multipotent MSCs has been found to inhibit the invasion of Fusobacteria in the oral cavity [64]. It has also been reported that the infusion of both hucMSC-Exs and hFP-Exs reduces the abundance of proinflammatory intestinal bacteria such as Verrucomicrobia and *Akkermansia muciniphila* to improve colitis [65].

#### 3.1.2. Increase in Beneficial Flora

In healthy humans, more than 90% of the gut microbiota are Firmicutes and Bacteroidetes [13]. Firmicutes and Bacteroidetes can metabolically produce short-chain fatty acids (SCFAs) [66]. SCFAs, especially butyrate, have been shown to induce the differentiation of regulatory T cells and maintain intestinal homeostasis [67]. Several studies have demonstrated the anti-inflammatory properties of Bacteroidetes [68,69], and inhibiting this species could lead to intestinal inflammation. However, most patients with IBD have a decrease in both Firmicutes and Bacteroidetes. MSCs are able to upregulate the ratio of Firmicutes and Bacteroidetes and increase the abundance of healthy flora such as *Lactobacillus murinus* and *Lactobacillus johnsonii* in a mouse model of IBD [70].

Furthermore, probiotics such as the Bifidobacterium, Lactobacillus, and Faecalibacterium genera can alleviate intestinal inflammation by modulating the release of cytokines, including the down-regulation of inflammatory cytokines, as well as promoting the production of IL-10 [71,72]. Recent studies have elucidated the role of probiotics in CD [73] and found that they are able to alleviate intestinal inflammation, although there is uncertainty about their efficacy. Bifidobacterium and Lactobacillus can also have a therapeutic role by repairing the intestinal barrier in IBD zebrafish [74]. In IBD, these probiotics are significantly reduced, perpetuating inflammation [75]. High-throughput sequencing (16rRNA) of DSS-induced IBD mice treated with MSC-Exs showed that the MSC-Ex treatment reversed a colitis-induced decrease in OTUs, Lactobacillus, and Bacteroides [76].

Defects in the mucus gel (MGL) layer are common in intestinal inflammation, and this defect leads to direct bacterial contact with the colonic surface, allowing bacterial invasion and the disruption of intestinal homeostasis [77]. Akkermansia, associated with MGL formation, is significantly reduced in IBD [78]. Recent studies have shown that the treatment of BALB/c mice with MSCs and endothelial progenitor cells (EPCs) results in positive gut flora alterations, accelerated mucosal damage repair, and increased Akkermansia [79].

#### 3.1.3. Enrichment and Balance of Intestinal Microbiota

Dysbiosis of the gut microbiota in IBD patients is not only due to an increase in harmful flora, as well as a decrease in beneficial flora, but also, more importantly, a decrease in the diversity of the flora, which presents a situation in which certain types of flora are prominently featured while others are virtually absent [80]. MSC-Exs significantly restore the structure of OTUs, decrease alpha diversity induced by colitis, and improve the composition of the intestinal microbiota [81]. Do-Wan Kim et al. found that MSCs could be delivered to intestinal crypts with stem-cell-loaded hydrogel microcapsules (SC-HMs) and modulated the intestinal microbiota in an IBD model in mice, including *Bacteroides acidifaciens*, *Lactobacillus* (*L.*) *gasseri*, *Lactobacillus reuteri*, and *L. intestinalis*, among other strains in dysbiosis, resulting in an increase in the abundance of gut microbiota [82]. In TNBS-induced colitis mice, MSC restores the normal characteristics of the gut microbiota; increases α-diversity; and increases the content of Bacteroidetes, Firmicutes, and Tenericutes while also decreasing the number of Proteobacteria [83].

MSC can also regulate gut microbiota dysbiosis in mouse models of other diseases, where a disease-related gut microbiota increases, along with a decrease in the immunomodulatory flora, which can be reversed by MSC treatment, possibly due to the involvement of MSCs in changing some metabolic pathways of the gut microbiota [84].

### 3.2. Mechanisms of MSCs in Regulating Gut Microbiota

#### 3.2.1. Directly Affecting Specific Strains

SCFAs produced by intestinal bacteria have positive effects on IBD. For example, butyrate can reduce the aggregation of neutrophils, thus ameliorating DSS-induced colitis in mice [85]. Recently, it was shown that butyrate can also ameliorate increased intestinal epithelial permeability induced by AIEC pathobionts (e.g., strain LF82) in UC and maintain the normal morphology and function of epithelial cell mitochondria [86]. Huc-MSCs can upregulate the levels of SCFA-producing bacteria, including Akkermansia, Faecalibaculum, and Clostridia_UCG_014, in the IBD model, which, in turn, promotes T cell homeostasis, thereby alleviating the inflammatory state of the intestinal mucosa [87]. HucMSC-Exs are similarly able to increase the levels of SCFAs, especially butyrate, which upregulates bacteria such as Bacteroides, *Parabacteroides distasonis*, and Tannerellaceae [88].

The gut microbiota is an important component of bile acid metabolism, hydrolyzing and dehydroxylating primary bile acids into secondary bile acids (SBAs). In the literature, SBAs and the SBA-producing bacteria rumenococcaceae have been significantly reduced in colitis mice, and intestinal inflammation has been reduced when SBAs have been given to colitis mice. SBAs can bind to the downstream anti-inflammatory receptor signals FXR and TGR5 to exert anti-inflammatory effects [89,90].

The FXR receptor is an important target for the interaction between intestinal microorganisms and the host immune system; thus, the ability of MSC-Exs to regulate its expression is a crucial therapeutic target in IBD [91]. By assessing the modulatory effect of MSC-Exs on gut bacteria composition and diversity and metabolites and their related functions and pathways in IBD, a study found that MSC-Exs modulate the gut metagenomic–metabolomic profile and increase the colonic FXR receptor [81]. This suggests that MSCs may exert their effects on microorganisms associated with IBD through the FXR pathway. In one study, in a mouse model of DSS-induced IBD, the intraperitoneal injection of hucMSCs increased the number of regulatory T cells in the gut-associated lymphoid tissue and elevated the level of immunoglobulin A, playing an anti-inflammatory role. The authors concluded that hucMSCs ameliorate DSS-induced colitis by not only regulating the Tregs-IgA response and enhancing the secretion of IgA but also by promoting the restoration of intestinal microbiota [92]. SIgA binds intestinal commensal bacteria, preventing them from passing through the intestinal barrier [93]. Consequently, MSC-Exs have the potential to improve dysbiosis by increasing the release of SIgA. Furthermore, miR-181a in MSC-Exs can alleviate a DSS-induced colitis model and restore its gut microbiota to a healthy state [76]. In another study, miR-150-3p in MSC-Exs was found to regulate the TRAF6/NF-κB axis and gut microbiota, thereby improving a mouse ICH (intracerebral hemorrhage) model [94]. At present, there is a paucity of research investigating the related molecules and signaling pathways between MSC-Exs and gut microbiota. This provides a foundation for future research on the specific mechanisms of MSC-Ex action on gut microbiota.

#### 3.2.2. Indirectly Affecting the Microbiota by Modulating the Host’s Immune Response

HucMSC-Exs maintain immune balance by regulating immune cells, which, in turn, improves the structure of intestinal epithelial cells, thereby improving the intestinal microenvironment on which the gut microbiota depends. They have a beneficial effect on the restoration of the gut microbiota [95]. It has been extensively reported that MSCs achieve immunomodulatory functions through the release of several cytokines [96,97], of which the most likely involved in the inflammatory regulation of MSCs are TNF-α-stimulated gene/protein 6 (TSG-6), as strong evidence indicates that TSG-6-deficient MSCs fail to improve colitis [98]. In 2018, Woo-Jin Song further conducted a study on the mechanism of TSG-6, where DSS-colitis mice treated with MSCs had significantly reduced TNF-α and IL-6 but increased IL-10, and the down-regulation of TSG-6 decreased the anti-inflammatory effect of MSCs. Since macrophages mainly secrete these cytokines, the coculture of macrophages with cAT-MSCs (canine adipose tissue-derived MSCs) showed that TSG-6 produced by cAT-MSCs could induce the conversion of M1 macrophages into M2 macrophages in vitro. Also, by assessing the number and phenotype of macrophages in an inflamed colon, it was found that the percentage of total macrophages was significantly lower in the cAT-MSC-treated group compared with the control group, while the percentage of M2 macrophages was significantly increased [99]. Similarly, MSC-Exs regulate Th2 and Th17 cells in mesenteric lymph nodes (MLNs) and repair the intestinal barrier by targeting TSG -6, significantly improving the structure and function of damaged intestinal epithelial cells [100]. Recent studies have found that the conversion of M1 cells into M2 cells by MSCs and, thus, their immunomodulatory effects can be mediated by EVs [101], while miRNA in MSC-Exs plays a major role [102]. MSC-Exs play a role in the treatment of IBD by down-regulating the inflammatory response by activating M2 macrophages. Multiple proteins are involved in this process in MSC-Exs, among which, metallothionein-2 is essential [103].

**Figure 3:**
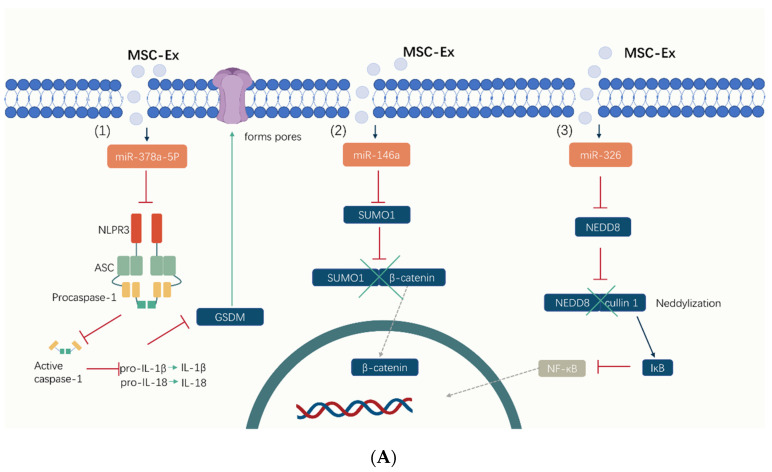

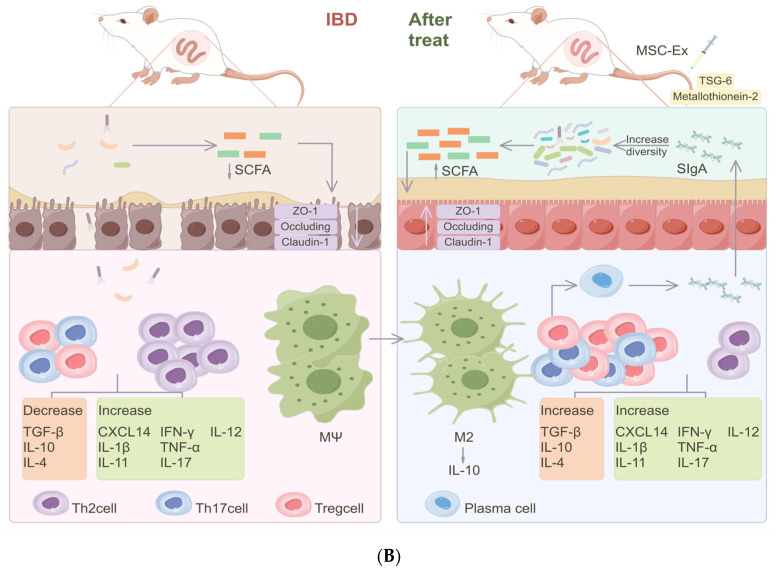
The mechanisms of MSC-Exs in the treatment of IBD. (**A**) MSC-Exs work by releasing small molecules like (1) miR-378a-5p, (2) miR-326, (3) miR-146a, and proteins. MSC-Exs modulate intracellular signaling pathways (including inhibiting cell focalization, ubiquitination, and neddylation) by influencing protein expression levels, thereby reducing inflammation. (**B**) MSC-Exs improve the diversity of the gut flora and its products, like SCFAs. By releasing metallothionein-2, TSG-6, and other important proteins, MSC-Exs regulate the number and function of T cells and activate macrophages (M2). Cytokines are also altered in this manner, with an increase in anti-inflammatory cytokines, reducing inflammation and, thus, improving the intestinal barrier.

MSC-Exs regulate various cytokines and Treg cells, increase the level of anti-inflammatory factors, reduce the level of proinflammatory factors, restore the balance of Th17/Treg cells, and reduce intestinal inflammation (Figure 3) [103]. Treg cells are an important class of immune cells that maintain immune homeostasis and self-tolerance, and the excessive activation of Treg cells in IBD patients causes damage to the intestinal mucosa. One study investigated T cell regulation by hucMSC-Exs and fetal placenta (FP)-MSCs and found that, while DSS induces a significant increase in Treg cell levels in mice, hucMSCs-Ex and FP-Ex treatments decrease Treg levels. Also, the MSC-Exs could regulate the concentration and expression of cytokines, as IL-10, IFN-γ, IL-14A, and IL-7 were significantly reduced in the peripheral blood of DSS-treated mice but markedly restored after treatment with hucMSCs-Exs or FP-Exs. Thus, MSC-Exs can restore immune homeostasis to the inflammatory microenvironment of the intestine [65]. Moreover, another study analyzed the number and proportion of T cells in the spleens and MLNs of mice with colitis after treatment with hucMSCs and found that hucMSCs inhibit apoptosis, promote Type 1 regulatory T (Tr1) cell proliferation, increase the proportion of Tr1 cells, and enhance the immunosuppressive function of Tr1 based on their paracrine indoleamine-2,3- dioxygenase (IDO), and when IDO is blocked, MSCs are unable to upregulate Tr1 cells [104].

#### 3.2.3. Repair of the Intestinal Barrier

MSCs and their derived exosomes potently regulate the function of intestinal epithelial cells (IECs). IECs line the small intestine and large intestine and play an important role in the digestion and absorption of nutrients. MSCs and their derived exosomes have been shown to exert several beneficial effects on IECs, including protection from injury, increased proliferation and differentiation, and reduced inflammation [105]. One study treated isolated mesenteric cells with MSCs and found that MSCs induce IEC-6 cell proliferation, reduce IEC-6 cell apoptosis, and enhance their migration. Hypoxia-pretreated MSCs further enhance these effects, which may be related to the activation of the PI6-Akt pathway in IEC-3 induced by hypoxia-pretreated MSCs [96]. Jingling Su et al. found the same supportive effect of MSCs on IEC-6 cells by culturing them in an MSC-containing DMEM medium. In addition to this, they found increased viability in IEC-6, and these promotive effects could be enhanced by IL-25, with the PI3K-Akt pathway playing a key role [106]. Recently, Peng Liu et al. also reported that MSC-CM pretreated with 25 μM of H2O2 can also enhance the repair effect of MSCs on the intestinal mucosa, with the activation of the Nrf2/Keap1/ARE pathway playing a key role [107]. In another study that explored the mechanism of MSC-Exs carrying microRNA-378a-3p (miR-378a-3p) to target IBD, the authors used bioinformatics analysis to identify GATA-binding protein 2 (GATA2) as a target gene of miR-378a-3p that regulates inflammation. Further, an analysis of related gene expression in the colonic mucosa of IBD patients revealed that miR-378a-3p could inhibit the GATA2/AQP4/PPAR-α pathway, thereby reducing apoptosis in mouse colonic epithelial cells [108]. Jun Xu et al. performed an RNA sequencing analysis of mouse colon tissue in a DSS colitis model and found that the upregulated DEGs (significantly differentially expressed genes) in the MSC group were mainly enriched in the annotation of intact components of the membrane. Thus, MSCs play a key role in maintaining colon cell integrity. Further analysis has revealed that this role is associated with significantly higher IGF-1 in serum after MSC treatments, which, in turn, upregulates the IGF1R-PI3K-AKT pathway and maintains colonic epithelial cell integrity [109]. MSCs also improve the secretory function of the intestinal mucosa; a study investigating the expression of the intestinal mucosal mucin 5ac found that 5ac is significantly reduced in DSS mice but increased in the colonic mucosa of MSC-treated mice compared with blank controls, and MUC8ac increased 5-fold in MSC-Ex-treated mice [110].

The normal intestine has intact tight junctions and bridging granules, but in mice with necrotizing small intestinal colitis, the tight junctions are open, and the adnexal epithelial cells are separated. However, the tight junctions in MSC-treated intestines are essentially normal, and the epithelial cells are regularly arranged, indicating that MSCs can significantly improve the tight junctions [111]. Tight junction proteins are important components of junctions, including zonula occludens-1 (ZO-1), Occludin, and Claudins. The ZO-1 protein is often used to assess the usual integrity of the intestine, and its expression or decreased levels can prevent the formation of tight junctions and cause intestinal inflammation. MSCs restore ZO-1 protein levels in DSS-induced colitis [112]. An experiment investigating the therapeutic mechanism of MSC-Exs in a rat model of intestinal ischemia–reperfusion (I/R) injury (IIRI) found that MSC-Exs increase Claudin-3, Claudin-2, and ZO-1 levels in Caco-2 cells and improve intestinal epithelial tight junctions. Further studies have revealed that this is mediated by miR-34a/c-5p and miR-29b-3p targeting the 3′ untranslated region (3′UTR) of the Snail transcription factor [113]. Similarly, Yi-Jun Li found that miR-34a-5p also causes an increase in tight-junction-related proteins such as ZO-1, Occludin, Zonulin, and Claudin-3, and an analysis of the upstream signaling of miR-34a-5p revealed that METTL3/IGF2BP3-mediated m6A modification causes MSC-Exs to secrete miR-34a-5p levels, thereby upregulating the tight junction proteins [114]. Moreover, studies have shown that MSCs and MSC-Exs ameliorate the massive deposition of collagen in the colon submucosa of DSS mice [107,110,112].

The intestinal barrier is capable of resisting microbial invasion, and IBD patients have an impaired intestinal barrier, including damage to the intestinal epithelium and disruption of tight junctions. Microbial infiltration into the intestinal mucosa can trigger a detrimental cascade, leading to an adverse intestinal environment. MSC can maintain the intestinal barrier in many ways, and a structurally and functionally intact intestinal mucosal barrier maintains intestinal homeostasis and prevents intestinal inflammation.

## 4. Prospects and Challenges of MSCs and MSC-Exs in IBD Therapy

### 4.1. Efficacy in Animal Models of IBD and Clinical Trials

Multiple experiments have shown that MSCs and their derived exosomes can effectively relieve experimentally induced colitis in mice (Table 1). In one such study, mice with DSS-induced colitis were treated with MSCs, MSC-Exs, and placebo in different groups, and the weight loss, stool viscosity, and hematochezia of the mice were recorded and analyzed. It was found that both MSCs and MSC-Exs could relieve colitis in mice and had the same inhibitory effect on inflammation [115]. Moreover, by studying the short-term and long-term protective effects of MSC on experimental colitis, a study found that MSCs derived from human adipose tissue not only relieve experimental colitis in the short term but also have long-term beneficial regulatory effects on IBD [116].

It was reported that among more than 200 patients with refractory fistulas who received a local injection of MSCs, more than half of the patients had complete remission. In 49 reported cases of refractory luminal CD who received systemic bone marrow MSC infusions, the patients who received autologous MSCs had relieved clinical symptoms, while about 40% of those who received allogeneic MSCs were relieved [117]. In addition, a trial evaluating the safety and efficacy of MSC-Exs in treating refractory fistulas in patients with IBD showed that all five treated patients had no adverse effects, and three showed complete healing [118]. Although MSCs have positive therapeutic effects in many animal models, their effectiveness in clinical applications needs further studies (Table 2).

### 4.2. Limitations and Future Prospects of MSC/MSC-Ex Treatments in IBD

MSCs can treat IBD by restoring the gut microbiota, forming an inflammation-suppressing microenvironment and repairing damaged mucosa. MSCs present a potentially superior alternative to the usual therapies for IBD. However, when the recipient mice and the MSCs’ MHCI and MHCII are mismatched, the infusion of MSCs can cause immune rejection [131] and produce more serious consequences. In addition to the therapeutic advantages exhibited by MSCs, hucMSC-Exs are characterized by low immunogenicity and have not yet been associated with adverse events in IBD treatments.

However, most of the studies on MSCs and MSC-Exs are in the preclinical stage, with fewer and unrepresentative studies of clinical patients. Furthermore, the preparation processes for MSCs and MSC-Exs are laborious and time-consuming. Also, the extraction methods for MSC-Exs are different, and thus, extracts through different methods show heterogeneity [132]. Although MSC-Exs are often identified by the size of their microvesicles and surface markers [133], biomolecules of similar sizes to MSCs are often mixed in, making it difficult to obtain high-purity MSC-Exs. There is an urgent need to find a uniform and standardized production method to resolve this dilemma.

Different sources of MSCs have distinct biological properties, resulting in different therapeutic effects on IBD [134]. The composition of MSC-Exs is complex, and there is no unanimous conclusion yet. Most studies have characterized the miRNA content and identified its possible downstream pathways for alleviating intestinal inflammation, but the protein and lipid constituents have had far from sufficient investigations. There is a need to identify as many substances in MSC-Exs as possible, and the mechanism of action of these substances on IBD must be well explored in order to better utilize their therapeutic effects.

### 4.3. Optimizing the Route of Administration

In the study of MSCs and MSC-Exs for the treatment of IBD mice, the method of tail vein injection is generally used. In this mode of administration, the survival rate of MSCs is lower, and the retention time of MSC-Exs in the body is shorter [135]. A novel delivery method has been initially investigated and could enhance therapeutic outcomes by mimicking the ecological environment of natural stem cells through the use of a hydrogel matrix and growth factors as a carrier. This approach could greatly improve the survival rate of stem cells [136]. The co-transplantation of CS-IGF-1C hydrogel with human placenta-derived MSCs (hP-MSCs) could increase the colonization of hP-MSCs in the intestines of colitis mice and enhance the therapeutic effect of MSCs [137]. The oral delivery of stem-cell-loaded hydrogel microcapsules (SC-HMs) could also positively affect the colonization of MSCs in IBD mice [82]. A special hydrogel can also be used as a slow-release carrier to keep the exosomes persistently at the damaged site for a long-lasting effect [138]. The retention time of exosomes in vivo can also be significantly increased by the subcutaneous transplantation of Bio-GelMA@Bio-EX hydrogels [139]. Therefore, new technologies such as hydrogel carriers should be used in the future to optimize the therapeutic potential of MSCs and MSC-Exs. It is also necessary to develop more new technologies for delivering MSCs and MSC-Exs in combination with nanomedicine, materials science, and other disciplines to achieve the same effect on IBD as cell infusion alone to accelerate clinical progress.

## 5. Conclusions

MSCs and their exosomes have been shown to be a promising alternative therapy for the treatment of experimentally induced colitis in mice and have demonstrated efficacy in clinical treatment. The gut microbiota of mice treated with MSCs or MSC-Exs exhibit a decrease in harmful bacteria and an increase in beneficial bacteria, as well as an increase in species of bacteria. MSCs and MSC-Exs can treat IBD by regulating microbiota metabolism, the immune microenvironment, and intestinal barriers to improve intestinal functions. Nevertheless, there are certain constraints to the utilization of MSCs and MSC-Exs. It is anticipated that their clinical applicability can be enhanced by integrating novel technologies, such as hydrogels, in future studies.

## Figures and Tables

**Table 1:** The role of MSCs and MSC-Exs in IBD.

Disease	Treatment Given	Mode of Administration	Study Model	Results	Reference
IBD	I-MSCs, AD-MSCs	Tail vein injection	In vivo (mice)	Both iMSCs and adMSCs reduced intestinal lesion scores, restored intestinal epithelial integrity, and improved microbial dysbiosis.	[57]
IBD	MSC-Exs	Tail vein injection	In vivo (mice)	Infusion of MSC-Exs converted Treg and Th17 cells in colitis mice into maintain immune homeostasis. Reduced the abundance of proinflammatory intestinal bacteria to ameliorate colitis.	[65]
IBD	MSC-Exs	Intravenous infusion	In vivo (mice)/in vitro (HCOEPIC)	MSC-Exs reduced colonic inflammation; TNF-α, IL-6, IL-1β, IL-17, and IL-18 levels were decreased; Claudin-1, ZO-1, and IκB levels were increased. In addition, the structure of the intestinal microbiota of colitis mice was improved.	[76]
IBD	MSCs	Intraperitoneal injection	In vivo (mice)	MSCs alleviated colitis by modulating the dysregulation of metabolic pathways and normalizing the function of abnormal flora in colitis mice.	[83]
IBD	HucMSCs	Intraperitoneal injection	In vivo (mice)	HucMSCs improved gut flora and upregulated the abundance of SCFA-producing bacteria. They also remodeled T cell immune homeostasis, resulting in a decrease in Th17 and an increase in Th2 and Treg. This had the effect of alleviating colitis.	[87]
IBD	HucMSCs	Peritoneal injection	In vivo (mice)	HucMSC improved intestinal lesions. It caused a significant increase in the proportion of Tregs and plasma cells, resulting in elevated intestinal and fecal IgA levels. In addition, microbiome alterations in colitis mice were partially restored.	[92]
IBD	HucMSC-Exs	Peritoneal injection	In vivo (mice)	HucMSC-Ex attenuated visual and histological colitis lesions by modulating Treg/Th17 balance, increasing anti-inflammatory, and decreasing pro-inflammatory cytokine expression.	[95]
IBD	BM-MSCs	Intraperitoneal injections	In vivo (mice)	BM-MSCs formed aggregates in the peritoneum and produced the immunomodulatory factor TSG6, thereby reducing intestinal inflammation.	[98]
IBD	cAT-MSCs	Intraperitoneal injection	In vivo (mice)	cAT-MSC-secreted TSG-6 ameliorated IBD and regulated colonic expression of pro- and anti-inflammatory cytokines, inducing a shift in macrophage phenotype from M1 to M2 in mice.	[99]
IBD	HucMSC-Exs	Intraperitoneal injection	In vivo (mice)	MSC-Exs prevented IBD by restoring mucosal barrier repair and intestinal immune homeostasis via TSG-6 in mice.	[100]
UC	BMSC-Exs	Peritoneal injection	In vivo (mice)/in vitro (LPS-treated macrophages)	BMSC-Exs attenuated the inflammatory response, resulting in the down-regulation of pro-inflammatory and up-regulation of anti-inflammatory factors, and promoted macrophage conversion into M2.	[101]
IBD	ADMSC-Exs	Intraperitoneal injection	In vivo (mice)	adMSC-Exs may reduce the clinical manifestations of IBD by modulating Treg populations and cytokines.	[103]
IBD	MSCs	Intraperitoneal injections	In vivo (mice)	hUCMSCs increased the proportion of Tr1 cells in the spleen and mesenteric lymph nodes in colitis; decreased the proportion of helper T cells (Th1 and Th17 cells); promoted the proliferation of Tr1 cells; and inhibited apoptosis. Effective relief of IBD.	[104]
IBD	MSC-Exs (miR-378a-3p)	Intravenous infusion	In vivo (mice)/in vitro (IEC-6)	MSCs-Exs can inhibit IBD by reducing GATA2 expression and down-regulating AQP4 to block the PPAR-α signaling pathway	[108]
IBD	T-MSCs	Intravenous infusion	In vivo (mice)	Intravenous infusion of T-MSCs increased circulating IGF-1 levels and alleviated colitis in mice.	[109]
IBD	MSCs	Enemas	In vivo (mice)	MSCs may be effectively involved in intestinal mucosal repair in experimental colitis through activation of the Nrf2/Keap1/ARE pathway.	[112]

Abbreviations: I-MSCs: induced pluripotent stem cell-derived mesenchymal stem cells; AD-MSCs: adipose-derived mesenchymal stem cells; HucMSCs: human umbilical cord mesenchymal stem cells; BM-MSCs: bone marrow mesenchymal stem cells; cAT-MSCs: canine adipose tissue-derived mesenchymal stem cells; BMSC-Exs: exosome secreted by bone marrow mesenchymal stem cells; T-MSCs: MSCs from human embryonic stem cells.

**Table 2:** Clinical trials regarding the effects of MSCs on IBD and their complications.

Disease	Treatment Given	Number of Patients	Assessment Time	Result	Reference
Luminal CD	BM-MSC	15	42 days	(1) Reduced CDAI and CDEIS scores in patients with biotherapy-refractory luminal CD. (2) One patient developed a serious adverse reaction (probably not caused by MSCs).	[119]
PfCD	BM-MSC	21	24 months	(1) Injections of 3 × 10^7^ MSCs appear to promote healing of perianal fistulas. (2) There were no serious adverse effects.	[120]
PfCD	BM-MSC	13	4 years	(1) Fistula closure or reduction in size. (2) No adverse reactions.	[121]
CD strictures	BM-MSC	10	48 weeks	(1) Complete or partial regression of stenosis. (2) No adverse effects.	[122]
PfCD	BM-MSC	22	6 months	(1) Improved healing rates and decreased indices, PCDAI, Wexner incontinence score, and van Assche score. (2) No adverse effects	[123]
Pediatric perianal CD	BM-MSC	7 (13–17 years)	12 months	(1) In total, 83 percent of patients had complete healing. Decrease in PCDAI, Wexner incontinence score, and van Assche score. (2) No adverse effects.	[124]
UC	BM-MSC	6	3 months	(1) The Mayo endoscopic severity score decreased. (2) No adverse effects.	[125]
PfCD	AD-MSC	212	24 months	(1) Relieves complicated perianal fistulas in patients with Crohn’s disease. (2) Adverse effects such as anal abscesses and rectal pain have occurred.	[126]
PfCD	AD-MSC	37	48 weeks	(1) In total, 56% of patients achieved clinical remission. (2) Seven cases had serious adverse reactions.	[127]
PfCD	AD-MSC	16	48 weeks	(1) Effective treatment of fistulous perianal Crohn’s disease in half of the patients, and induced good MRI changes. (2) No adverse effects.	[128]
PfCD	UC-MSC	82	12 months	(1) The CDAI, HBI, and corticosteroid dosage were decreased. (2) There were minor adverse reactions (fever) and no serious adverse reactions.	[129]
PfCD	UC-MSC	10	52 weeks	(1) Significant improvement in PCDAI, pelvic MRI score, CDAI, and quality of life score and 70% relapse-free at 52 weeks. (2) No serious adverse effects.	[130]

Abbreviations: CD: Crohn’s disease; CDAI: Crohn’s disease activity index; CDEIS: Crohn’s disease endoscopic index of severity; PfCD: perianal fistulizing Crohn’s disease; PCDAI: perianal Crohn’s disease activity index; UC: ulcerative colitis; MRI: magnetic resonance imaging; HBI: Harvey–Bradshaw index.

## Data Availability

The data are contained within the article.
